# *SRSF1* haploinsufficiency drives the neurodevelopmental phenotype of the 17q22 deletion syndrome

**DOI:** 10.3389/fmed.2026.1750790

**Published:** 2026-04-10

**Authors:** Youlan Wu, Chuanfen Gao, Zhenghe Chen, Fang Liu, Jing Yuan, Weisheng Cheng

**Affiliations:** 1 Department of Obstetrics and Gynecology, Prenatal Diagnosis Center, The First Affiliated Hospital of Anhui Medical University, Hefei, China; 2 NHC Key Laboratory of Study on Abnormal Gametes and Reproductive Tract (Anhui Medical University), Hefei, China; 3 Engineering Research Center of Biopreservation and Artificial Organs, Ministry of Education, Hefei, China; 4 Department of Ultrasound, The First Affiliated Hospital of Anhui Medical University, Hefei, China; 5 Anhui Provincial Institute of Translational Medicine, Hefei, China; 6 Department of Clinical Laboratory, The First Affiliated Hospital of Anhui Medical University, Hefei, China; 7 Anhui Province Key Laboratory of Reproductive Disorders and Obstetrics and Gynecology Diseases, Hefei, China

**Keywords:** 17q22 deletion, craniofacial anomalies, neurological abnormalities, prenatal diagnosis, *SRSF1*

## Abstract

**Background:**

The 17q22 deletion syndrome is associated with a range of clinical phenotypes, primarily due to differences in the size and position of the deleted genomic segment. In all previously reported cases, diagnoses were made postnatally, with clinical manifestations such as intellectual disability, visual impairment, and other neurological abnormalities.

**Methods:**

Karyotype analysis, chromosomal microarray analysis, and whole-exome sequencing were performed to identify the genetic etiology of a fetus with nuchal pellucida thickening. Summarize the previously reported cases of similar 17q22 deletions and conduct a systematic review.

**Results:**

Chromosomal microarray analysis revealed an arr[GRCh37] 17q22(55,501,204_56,476,808) × 1, which was confirmed as a *de novo* variant by whole-exome sequencing. Comprehensive prenatal sonography revealed multiple structural abnormalities in the fetus, including neurodevelopmental abnormalities and distinct craniofacial features. A comprehensive search uncovered 24 postnatal cases with 17q22 deletions overlapping our findings. All these cases showed consistent neurological abnormalities, further supporting the genotype–phenotype association.

**Conclusion:**

Integrating the genetic findings in this fetus, the abnormal ultrasound structural phenotype, and the clinical characteristics of existing cases, we conclude that *SRSF1* haplotype deficiency is the fundamental genetic cause underlying the observed fetal ultrasound abnormalities. This report of the 17q22 deletion (include *SRSF1*) provides an essential reference for prenatal genetic counseling and phenotypic interpretation.

## 1 Introduction

The 17q22 deletion is a rare chromosomal aberration characterized by genomic imbalances in this region that are associated with a spectrum of complex clinical manifestations, including neurodevelopmental delays, psychiatric disorders, facial malformations, and skeletal or urogenital abnormalities (1). Microdeletion at 17q22 (include *NOG*) has been reported. The *NOG* gene encodes a bone morphogenetic protein antagonist that is essential for the development of bones, joints, and neural tubes (2, 3). *NOG* loss-of-function mutations cause skeletal disorders, including proximal interphalangeal joint fusion and multiple synostoses syndrome (4). However, it is worth noting that many cases of 17q22 deletion are also associated with neurodevelopmental phenotypes, including intellectual disability, developmental delay, attention-deficit and hyperactivity disorder, which cannot be explained by *NOG* haploinsufficiency alone, suggesting that other genes critical for neurodevelopment are present in this region.

In 2023, a study systematically characterized syndromic neurodevelopmental disorders arising from *SRSF1* haploinsufficiency (5). This finding implicates *SRSF1* as a core driver gene for the neurodevelopmental phenotype in 17q22 deletion. Notably, *SRSF1* and *NOG* are physically separated by >1 Mb, allowing *SRSF1* deletions to occur independently of *NOG* loss. Consequently, the phenotypic spectrum associated with *SRSF1* haploinsufficiency is distinctly different from the “classic 17q22 deletion” phenotype.

We report a fetus with chromosome 17q22 deletion syndrome (include *SRSF1*) presenting with abnormal ultrasound findings. We reviewed and statistically analyzed cases of overlapping 17q22 deletions to delineate the *SRSF1*-associated phenotypic spectrum. Current literature focuses on postnatal phenotypes, leaving a gap in evidence on prenatal detection and raising questions about the effectiveness of genetic counseling. Ultrasound-detected structural abnormalities often correlate with genetic anomalies (6–8). Therefore, this study presents a novel prenatal case of 17q22 deletion syndrome identified via ultrasound and genetic diagnosis, and conducts a systematic literature review to summarize the phenotypic spectrum and explore genotype–phenotype correlations, providing a reference for prenatal diagnosis and genetic counseling.

## 2 Methods

### 2.1 Clinical report

A 31-year-old pregnant woman, G2P1, reported no family history of genetic disease, consanguinity, drug use during pregnancy, or exposure to radioactive or toxic substances. The fetal ultrasound examination at 16 weeks revealed a thickened nuchal translucency (NT) measuring 4.3 mm, which warranted close monitoring and further diagnostic evaluation at a more advanced gestational age.

### 2.2 G-banding karyotype analysis

Amniotic fluid cells were cultured using the in-situ cell culture method, and chromosomes were prepared for karyotyping. Chromosomal karyotype analysis was performed at a 400-band level, strictly following the International System for Human Cytogenomic Nomenclature (ISCN 2024) (9).

### 2.3 Chromosomal microarray analysis

A total of 15 mL of amniotic fluid and 2 mL of maternal peripheral blood were collected. Following exclusion of maternal cell contamination by quantitative fluorescent PCR (QF-PCR), CMA was performed using the CytoScan 750 K Arrays (Biosan, China). Copy number variations (CNVs) results were cross-validated by integrating single-nucleotide polymorphism (SNP) probes and CNV-specific probes. Pathogenicity interpretation of CNVs was conducted in accordance with the Clinical Guidelines (10).

### 2.4 Whole-exome sequencing analysis

Amniotic fluid and peripheral blood from the pregnant woman and her spouse were collected. DNA was extracted using the Tiangen Biotech DNA Extraction Kit, followed by library preparation, quality control, and high-throughput sequencing. The raw sequencing data underwent quality control, alignment, variant calling, filtering, and annotation, and genetic pathogenicity analysis was conducted based on the processed data.

### 2.5 Similar cases review

In September 2025, we searched PubMed, DECIPHER (11), and the China National Knowledge Infrastructure (CNKI) for cases with 17q22 deletions using the keyword “17q22.” Inclusion criteria were: full-text availability, human clinical and genetic data, and deletions overlapping or closely aligning with our case. All eligible publications were reviewed and data systematically extracted.

## 3 Results

### 3.1 Ultrasound examination

**Figure 1:**
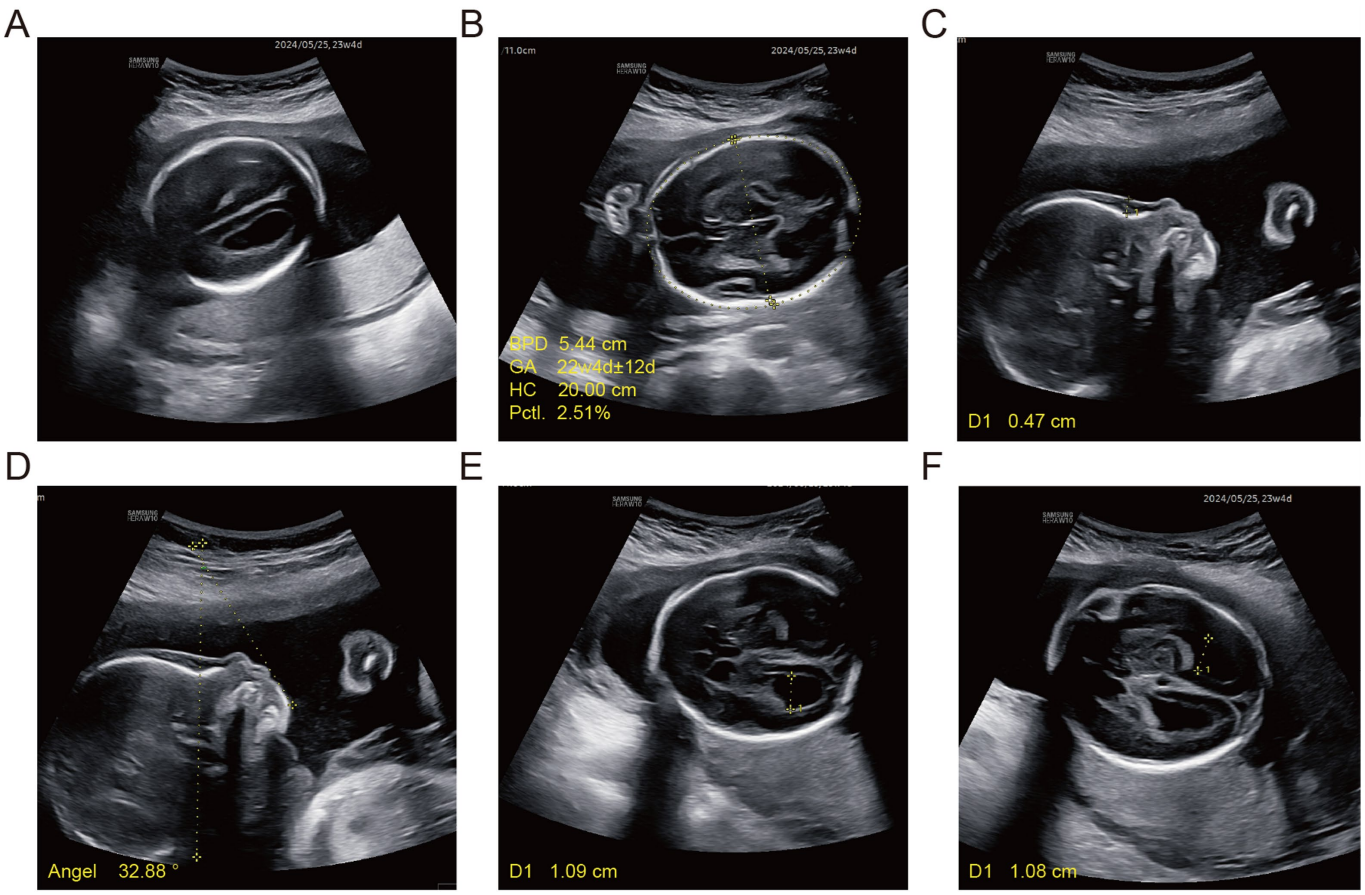
Fetal ultrasonography findings. **(A)** Bilateral parieto-occipital sulci are not visualized on the transcranial axial view. **(B)** Transthalamic axial plane: BPD (5.44 cm) and HC (20.00 cm) both < expected for gestational age. **(C)** Thickening soft tissue. **(D)** Frontal soft tissue thickening with a mandibular-facial angle <49° and micrognathia. **(E)** Right lateral ventricle posterior horn 10.9 mm (far-field standard plane). **(F)** Left lateral ventricle 10.8 mm (non-standard near-field imaging plane).

To track fetal growth and development and structure of various systems, fetal ultrasound examination at 23 weeks showed fetal biological parameters, including growth parameters lower than China gestational age reference standard, mild bilateral ventricular enlargement, no visible parieto-occipital sulcus, micrognathia, mild frontal lobe flat with frontal lobe soft tissue thickening, trace tricuspid regurgitation, and enlarged foramen ovale (Figure 1).

### 3.2 Genetic analysis

**Figure 2:**
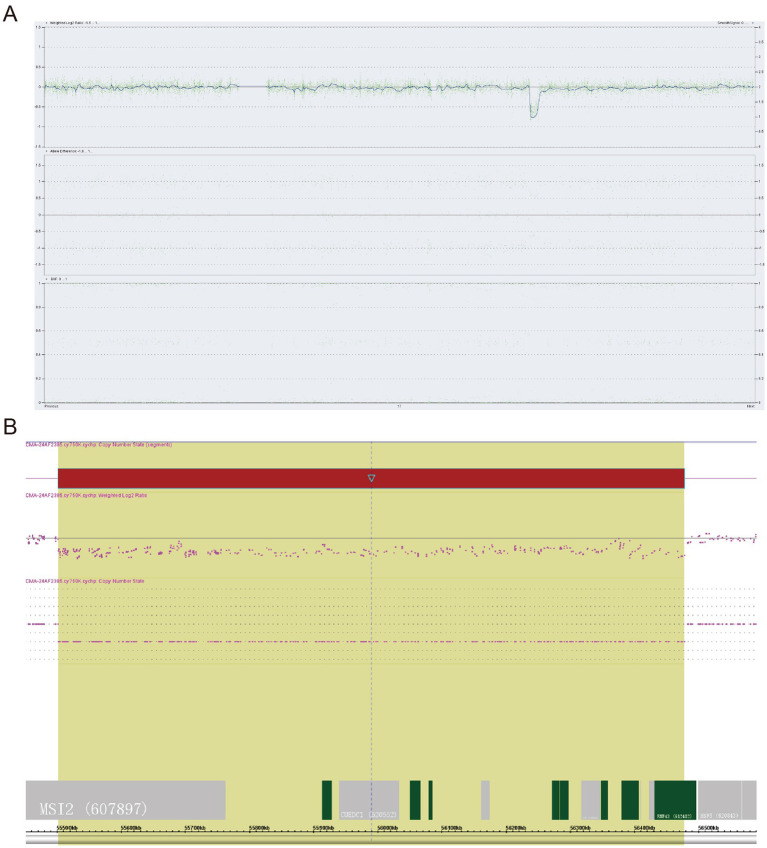
Deletion localization and identification by CMA. **(A)** Fragment copy deletion in region 17q22. **(B)** CMA identified the 17q22 deletion variant.

Fetal amniotic fluid G-banding karyotype was 46, U. with no detected abnormalities. The CMA result indicated arr[GRCh37] 17q22(55,501,204_56,476,808) × 1dn (Figures 2A, B). The results showed that there was a deletion of 976Kb in the 17q22 segment in this sample, involving 15 protein-coding genes such as *SRSF1*, *MKS1*, *VEZF1*, *DYNLL2*, *MPO*, spanning the exon 7–14 region of the *MSI2* gene (NM_138962) and the exon 3–10 region of *the RNF43* gene (NM_017763). WES results confirmed the fetal CMA finding, and the variant was not detected in either parent’s peripheral blood.

### 3.3 Cases search results

**Figure 3:**
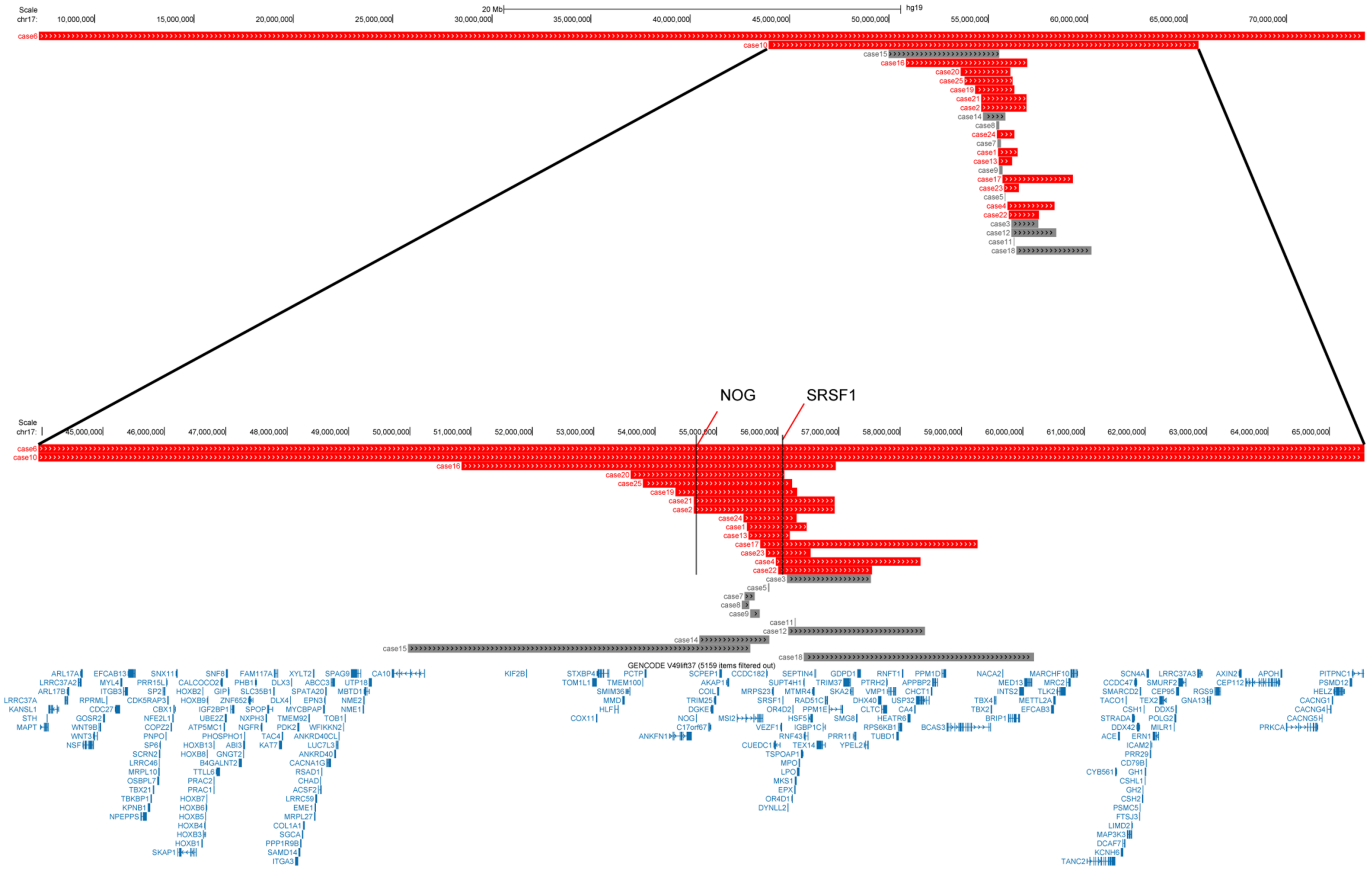
UCSC track summaries 17q22 deletion cases.

Cases were screened to find those describing deletions overlapping or adjacent to the chromosomal segment relevant to our case. A total of 24 previously reported cases met these criteria: 9 from PubMed, 14 from the Decipher database, and 1 from CNKI (Figure 3 and Table 1).

**Table 1:** Reported CNVs in similar chromosomal regions and corresponding phenotype.

Case	Genomic change (GRCh37)	Size	Location (GRCh38)	Include *SRSF1*	Include *VEZF1*	Include *NOG*	Other diagnosis	Neurological abnormalities	Craniofacial anomalies	Cardiovascular anomalies	Other systemic abnormalities	Reference
1	17q22(55,501,204_56,476,808) × 1	975.6 kb	chr17:57,423,843–58,399,447	Yes	Yes	No		Mild bilateral lateral ventriculomegaly, Non-visualized parieto-occipital sulcus	Mild frontal flattening, Thickened frontal soft tissue, Micrognathia	Tricuspid regurgitation, Enlarged foramen ovale		This study
2	17q22(54,638,141_56,925,730) × 1	2.29 Mb	chr17:56,560,780–58,848,369	Yes	Yes	Yes		Delayed speech and language development, Intellectual disability, Microcephaly	High palate, Long face		Camptodactyly of finger, Micropenis, Cryptorchidism, Chordee	Decipher:2292
3	17q22(56,155,740_57,518,153) × 1	1.36 Mb	chr17:58,078,379–59,440,792	No	No	No		Hypotonia			Feeding difficulties in infancy, Short stature	Decipher:248299
4	17q22q23.2(55,956,435_58,332,581) × 1	2.38 Mb	chr17:57,879,074–60,255,220	Yes	Yes	No		Intellectual disability	Facial dysmorphisms			Decipher:250690
5	17q22(55,842,501_55,858,958) × 1	16.46 kb	chr17:57,765,140–57,781,597	No	No	No		Cognitive impairment				Decipher:266762
6	17p13.1q25.1(7,206,144_73,956,430) × 1	68.66 Mb	chr17:7,302,825–75,960,349	Yes	Yes	Yes		Cerebral palsy, Generalized myoclonic seizure, Moderate intellectual disability, Polymicrogyria, Tetraplegia				Decipher:283428
7	17q22(55,464,726_55,625,219) × 1	160.49 kb	chr17:57,387,365–57,547,858	No	No	No		Intellectual disability	Ptosis		Short stature	Decipher:288846
8	17q22(55,413,841_55,536,131) × 1	122.29 kb	chr17:57,336,480–57,458,770	No	No	No			Anterior creases of earlobe		Large for gestational age, Macroglossia, Neonatal hypoglycemia	Decipher:354973
9	17q22(55,553,385_55,709,366) × 1	155.98 kb	chr17:57,476,024–57,632,005	No	No	No		Cognitive impairment, Language impairment			Congenital hypothyroidism	Decipher:378963
10	17q21.31q24.2(43,944,211_65,569,538) × 1	21.71 Mb	chr17:45,866,845–67,573,422	Yes	Yes	Yes		Intellectual disability, Proportionate short stature, Global development delay, Microcephaly	Brachycephaly, Long philtrum, Micrognathia, Plagiocephaly, Downturned corners of mouth, Hypertelorism, Round face, Short palpebral fissure, Telecanthus, Upturned palpebral fissure		Bifid uvula, Polyhydramnios, Redundant neck skin, Skeletal anomalies, Tracheoesophageal fistula	Decipher:395112
11	17q22(56,283,914_56,283,942) × 1	29 bp	chr17:58,206,553–58,206,581	No	No	No	MSK1:c.417G > A (p. E139E)	Chiari malformation, Hydrocephalus				Decipher:400388
12	17q22q23.2(56,174,379_58,402,200) × 1	2.23 Mb	chr17:58,097,018–60,324,839	No	No	No		Microcephaly			Overlapping fingers, Overlapping toes	Decipher:456208
13	17q22(55,529,533_56203785) × 1	674.25 kb	chr17:57,452,172–58,126,424	Yes	Yes	No						Decipher:476669
14	17q22(54,726,037_55,863,971) × 1	1.14 Mb	chr17:56,648,676–57,786,610	No	No	No					Esophageal atresia, Pancreatic cysts, Radioulnar synostosis	Decipher:487065
15	17q22(54,726,037_55,863,971) × 1	5.58 Mb	chr17:56,648,676–57,786,610	No	Yes	Yes		Microcephaly, Neurodevelopmental delay	Thin vermilion border, Triangular face			Decipher:526240
16	17q22(49,971,133_55,553,444) × 1	6.10 Mb	chr17:51,893,773–57,476,083	Yes	Yes	Yes		Intellectual disability, Microcephaly, Hearing impairment, Visual impairment, Global developmental delay	Trigonocephaly, High forehead, Upslanting palpebral fissures, Ptosis, Telecanthus, Epicanthal folds, Broad nasal root, Broad nasal tip, Thin upper lip, Mild maxillary hypoplasia		Cryptorchidism, Micropenis, Skeletal anomalies	(17)
17	17q22q23.2(55,717,537_59,257,880) × 1	3.54 Mb	chr17:57,640,176–61,180,519	Yes	Yes	NO		Ventriculomegaly, Macrocephaly, Hearing impairment, Visual impairment	Narrow palpebral fissures, Telecanthus, Low-set ears, Downturned corners of the mouth, Micrognathia	Tricuspid regurgitation	Micrognathia, Skeletal anomalies	(18)
18	17q22q23.2(56,429,075_60,181,763) × 1	3.75 Mb	chr17:58,351,714–62,104,402	No	No	No		Microcephaly, Hearing impairment, Visual impairment, Small posterior fossa, Global development delay, Conical cerebellar tonsils	Wide nasal bridge, tented upper lip, Wide mouth and mild synophrys, Simplified and posteriorly rotated ears with overfolded helices		Skeletal anomalies, Thyroglossal dust cyst	(19)
19	17q22(54,329,389_56,314,137) × 1	1.98 Mb	chr17:56,252,028–58,236,776	Yes	Yes	Yes		Microcephaly, Intellectual disability, Hearing impairment, Visual impairment	Micrognathia, Epicanthal folds, Bulbous nose, High nasal bridge, Narrow palpebral fissures, Telecanthus, Prominent and thin upper lip, Short and up-slanting palpebral fissures, Short philtrum	Aortic hypoplasia and a persistent ductus arteriosus	Skeletal anomalies	(1)
20	17q22q23.2(53,609,015_56,109,938) × 1	2.50 Mb	chr17:55,531,654–58,032,577	Yes	Yes	Yes		Intellectual disability, Speech delay, Visual impairment	Long, narrow face, Maxillary hypoplasia, Bulbous nose, Prominent columella, Telecanthus, Hypoplastic alae nasi, High nasal bridge, Short philtrum, Thin upper lip, Narrow palpebral fissures, Epicanthal folds, Preauricular pit		Skeletal anomalies	
21	17q22(54,638,141_56,925,730) × 1	2.29 Mb	chr17:56,560,780–58,848,369	Yes	Yes	Yes	1p13.2(113,036,203-113,159,136) × 3	Intellectual disability, Speech delay, Microcephaly	Long and narrow face, High-arched palate		Micropenis, Skeletal anomalies	
22	17q22(56,012,009_57,541,627) × 1	1.53 Mb	chr17:57,934,648–59,464,266	Yes	Yes	No	1p31.1(79,890,343-80,350,309) × 1	Intellectual disability, Global development delay, Speech delay	High nasal bridge, Upturned nasal tip, Smooth philtrum, High-arched palate, Large and posteriorly rotated ear, Thin upper lip		Skeletal anomalies	(20)
23	17q22(55,806,534_56,540,597) × 1	734.06Kb	chr17:57,729,173–58,463,236	Yes	Yes	No		Intellectual disability, Global development delay, Speech delay	Facial dysmorphisms	Tricuspid regurgitation	Skeletal anomalies	(5)
24	17q22(55,442,363_56,309,063) × 1	867.70Kb	chr17:57,365,002–58,231,702	Yes	Yes	No	15q11.2 BP1-BP2 microdeletion	Intellectual disability, Global development delay, Speech delay	Facial dysmorphisms		Genu varum	
25	17q22(53,806,777_56,233,636) × 1	2.24 Mb	chr17:55,729,416–58,156,275	Yes	Yes	Yes		Intellectual disability, Global development delay, Speech delay, Microcephaly	Telecanthus, Narrow palpebral fissures, Low-set ears, Thin upper lip, Downturned corners of the mouth, High-arched palate, Micrognathia, Broad nasal root	Tricuspid regurgitation	Skeletal anomalies	(21)

## 4 Discussion

Current reports of 17q22 deletion are mainly in pediatric and adult populations, with prenatal cases exceedingly rare. As ultrasonography is the primary tool for detecting fetal structural anomalies, characterizing the prenatal sonographic phenotypes associated with 17q22 deletion—alongside underlying genetic mechanisms—will provide essential diagnostic references and inform future research into the developmental pathogenesis of this disorder. In this case, NT thickening was detected by fetal ultrasound screening during early pregnancy. Genetic diagnostic investigations identified a *de novo* CNV. The segment contains 15 protein-coding genes, including *SRSF1*, *MKS1*, *VEZF1*, and *MPO*. *VEZF1* and *SRSF1* demonstrate elevated haploinsufficiency scores and were predicted to exhibit haploinsufficiency at the gnomAD database (Accessed September 20, 2025) (12).

*SRSF1* encodes a serine/arginine-rich splicing factor that interacts with other components of the spliceosomal complex to prevent exon skipping, thereby ensuring splicing fidelity and regulating alternative splicing (13). Haploinsufficiency of *SRSF1* shows a strong association with syndromic neurodevelopmental abnormalities characterized by intellectual disability (5), the primary manifestations include speech delay, behavioral abnormalities, and non-specific craniofacial anomalies. Analysis of the gnomAD database revealed marked intolerance to loss-of-function (LoF:pLI = 1, o/e = 0.04 (0.01–0.17) < 0.35). The Clingen database (Accessed September 20, 2025) (14) recognized *SRSF1* as a dose-sensitive gene, with haploinsufficiency linked to neurodevelopmental abnormalities, including dysmorphic facies and behavioral abnormalities. Current evidence indicates a direct link between *SRSF1* haploinsufficiency and fetal neurological and craniofacial anomalies.

A summary of cases from our case, databases and the existing literature reveals that among 15 cases with confirmed deletions encompassing *SRSF1*, the incidence of intellectual disability (ID) or global developmental delay (GDD) was 86.67% (13/15), indicating near-complete penetrance. The remaining two cases lacked assessment due to infant death or other reasons. A detailed breakdown shows that among the 12 cases explicitly documented with ID, 40.0% (6/15) also presented with GDD. Speech/language delay, another key indicator, was observed in 46.67% (7/15) of cases and frequently co-occurred with ID.

Among the 10 cases without *SRSF1* deletion, only 20.0% (2/10) presented with ID or GDD, both involving deletions of other neurodevelopmental candidate genes. The remaining cases with other neurodevelopmental phenotypes—such as cognitive impairment or nonspecific developmental delay—also all harbored deletions involving such candidate genes. These observations suggest that the neurodevelopmental manifestations in these cases are likely attributable to dosage alterations of genes other than *SRSF1* within the deleted intervals, rather than to *SRSF1* haploinsufficiency itself.

Craniofacial anomalies represent the second most characteristic feature after neurodevelopmental disorders, with an incidence of 80.0% (12/15), presenting a recognizable pattern. Although the specific manifestations are diverse, common elements include micrognathia (33.33%, 5/15), hypertelorism or epicanthal folds, thin upper lip vermilion, low-set ears, and a broad nasal root or bulbous nose. *SRSF1* haploinsufficiency appears to constitute a “necessary foundation” for both neurodevelopmental and craniofacial abnormalities. Microcephaly, a key quantifiable sign, was present in 40.0% (6/15) of cases. In-depth analysis revealed that these 6 microcephaly cases, the deletion interval encompassed both *SRSF1* and *NOG* in 100% (Figure 3 and Table 1). The crucial role of *NOG* in skeletal development might exacerbate the impact of *SRSF1* deletion on craniofacial morphology. When the deletion extends to include *NOG*, the phenotypic spectrum demonstrates an “additive effect”: on the foundation of core neurodevelopmental disorders, the incidence and distinctiveness of craniofacial anomalies—particularly microcephaly—and skeletal malformations become significantly more pronounced, culminating in a more complete and classic syndromic facies.

*VEZF1* is a key transcription factor essential for vascular system development, playing pivotal roles in vascular network formation, endothelial cell function, and the regulation of collagen deposition. Zou et al. (15) reported that *Vezf1* haploinsufficiency causes partial vascular disruption, suggesting 50% expression may support basic cardiovascular development. This is supported by Das et al., who showed that residual *VEZF1* synergizes with *ETV2* to activate Flt1, likely maintaining sufficient target gene expression for minimal requirements (16). In summary, haploinsufficiency of *VEZF1* may occur without manifesting overt structural cardiovascular phenotypes. In our case series, we found that all CNVs encompassing *SRSF1* invariably included *VEZF1*. Among these 15 co-deletion cases, cardiovascular anomalies were identified in 5 (33.33%), cases 17 and 25 exhibited patent ductus arteriosus, case 19 had aortic hypoplasia, and both cases 23 and our case presented with tricuspid regurgitation. The role of *VEZF1* in embryonic vascular development is well established, primarily through haploinsufficiency and regulation of protein interactions. The vascular network abnormalities observed in cases 17, 19, and 25 are strongly supported by evidence. Tricuspid regurgitation in cases 1 and 23 may result from *VEZF1*’s role in regulating early cardiovascular precursor cells in the embryonic mesoderm.

The physical proximity of *VEZF1* and *SRSF1* predisposes them to codeletion in 17q22 CNVs. *SRSF1* deletion drives intellectual disability and developmental delay, while *VEZF1* haploinsufficiency—a dosage-sensitive vascular transcription factor—likely causes cerebrovascular abnormalities, compromising neural blood supply and exacerbating neurological deficits. Their synergistic haploinsufficiency disrupts overlapping yet complementary pathways, contributing to the complex multisystem phenotype of 17q22 deletion syndrome.

Prenatal diagnosis is challenging and often involves rare cases, as ultrasound examinations frequently miss specific subtle structural abnormalities. Therefore, minor ultrasound anomalies play a significant role in indicating potential fetal abnormalities. In the present case, routine prenatal screening at 16 weeks of gestation revealed an NT thickening, raising suspicion of underlying chromosomal or structural defects. Although NT thickening is a non-specific soft marker with heterogeneous etiologies and cannot guide clinical decisions independently, the abnormal phenotypic findings provided a clear indication for invasive prenatal testing and comprehensive genetic analysis. Subsequent investigations identified a constellation of sonographic features—including fetal biometric parameters below Chinese reference standards for gestational age, mild ventriculomegaly, indistinct parieto-occipital sulcus, flattened frontal lobe, increased nuchal soft tissue, micrognathia, reduced frontal horn dimension, mild tricuspid regurgitation, and an enlarged foramen ovale—that were associated with a fetal copy number variant: a heterozygous deletion at 17q22 encompassing the *SRSF1* and *VEZF1* genes. To our knowledge, this is the first prenatal report of a 17q22 deletion syndrome (include *SRSF1*), providing novel insights into the genetic basis of a distinct prenatal ultrasound phenotype characterized by neurodevelopmental and craniofacial anomalies.

## Data Availability

The original contributions presented in the study are included in the article/supplementary material, further inquiries can be directed to the corresponding authors.
